# Progression in low‐intensity ultrasound‐induced tumor radiosensitization

**DOI:** 10.1002/cam4.7332

**Published:** 2024-07-05

**Authors:** Haonan Xu, Zichao Liu, Meng Du, Zhiyi Chen

**Affiliations:** ^1^ Key Laboratory of Medical Imaging Precision Theranostics and Radiation Protection, College of Hunan Province, The Affiliated Changsha Central Hospital, Hengyang Medical School University of South China Changsha Hunan Province China; ^2^ Institute of Medical Imaging, Hengyang Medical School, University of South China Hengyang Hunan Province China; ^3^ The Seventh Affiliated Hospital, Hunan Veterans Administration Hospital, Hengyang Medical School University of South China Changsha Hunan Province China

**Keywords:** bioeffects, low‐intensity ultrasound, mechanism, radiosensitization, tumor treatment

## Abstract

**Background:**

Radiotherapy (RT) is a widely utilized tumor treatment approach, while a significant obstacle in this treatment modality is the radioresistance exhibited by tumor cells. To enhance the effectiveness of RT, scientists have explored radiosensitization approaches, including the use of radiosensitizers and physical stimuli. Nevertheless, several approaches have exhibited disappointing results including adverse effects and limited efficacy. A safer and more effective method of radiosensitization involves low‐intensity ultrasound (LIUS), which selectively targets tumor tissue and enhances the efficacy of radiation therapy.

**Methods:**

This review summarized the tumor radioresistance reasons and explored LIUS potential radiosensitization mechanisms. Moreover, it covered diverse LIUS application strategies in radiosensitization, including the use of LIUS alone, ultrasound‐targeted intravascular microbubble destruction, ultrasound‐mediated targeted radiosensitizers delivery, and sonodynamic therapy. Lastly, the review presented the limitations and prospects of employing LIUS‐RT combined therapy in clinical settings, emphasizing the need to connect research findings with practical applications.

**Results and Conclusion:**

LIUS employs cost‐effective equipment to foster tumor radiosensitization, curtail radiation exposure, and elevate the quality of life for patients. This efficacy is attributed to LIUS's ability to utilize thermal, cavitation, and mechanical effects to overcome tumor cell resistance to RT. Multiple experimental analyses have underscored the effectiveness of LIUS in inducing tumor radiosensitization using diverse strategies. While initial studies have shown promising results, conducting more comprehensive clinical trials is crucial to confirm its safety and effectiveness in real‐world situations.

## Graphical abstract

This review summaries the tumor radioresistance reasons and explores LIUS potential radiosensitization mechanisms. Moreover, it covers diverse LIUS application strategies in radiosensitization, including the use of LIUS alone, ultrasound‐targeted intravascular microbubble destruction, ultrasound‐mediated targeted radiosensitizers delivery, and sonodynamic therapy. Lastly, the review presents the limitations and prospects of employing LIUS‐RT combined therapy in clinical settings, emphasizing the need to connect research findings with practical applications.

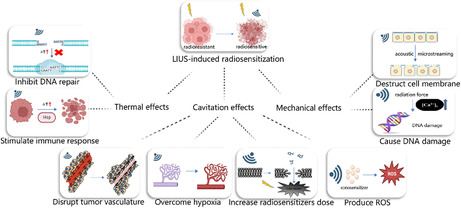

## 1 INTRODUCTION

Radiotherapy (RT) is a widely utilized tumor treatment method due to its ability to induce apoptosis in tumor cells by damaging DNA and promoting the production of free radicals.^1^ Over the past three decades, significant technological advancements have transformed the landscape of RT. The introduction of three‐dimensional image guidance, intensity modulation, and stereotactic body techniques has improved the precision of radiation delivery while minimizing damage to surrounding healthy tissues.^2^, ^3^ However, the development of radioresistance in tumor cells remains a challenge, compromising the effectiveness of RT.^4^ To overcome this obstacle, researchers have concentrated on the development of radiosensitization methods.^5^

Currently, radiosensitization methods include chemical radiosensitizers and physical stimuli. Chemical radiosensitizers encompass various agents, such as small molecules, macromolecules, and nanomaterials.^6^ These agents can enhance the killing effect on tumor cells by directly causing DNA damage and indirectly generating free radicals.^7^ Physical stimuli, including heat, light, and sound, have also been employed. Notably, the incorporation of hyperthermia and phototherapy has significantly improved RT outcomes.^8^, ^9^, ^10^ Despite these advances, challenges persist in the field of RT.^11^ Several existing radiosensitizers have limited water solubility and increased toxicity, which compromise their efficacy and amplify adverse effects.^12^, ^13^ Furthermore, light‐based techniques suffer from restricted tissue penetration depth, while heat poses risks to healthy tissue integrity.^14^, ^15^ These factors underline the need for pioneering innovative radiosensitization methods.

In the realm of physical stimuli, low‐intensity ultrasound (LIUS) has emerged as a promising method to enhance the efficacy of RT. LIUS is an output in low‐intensity pulse wave mode with an average sound intensity of less than 3 W/cm^2^, which has the least thermal effect while maintaining the transmission of sound energy to the target tissue and providing noninvasive physical stimulation.^16^ Compared with other methods, LIUS is a safer and more effective alternative. Its noninvasive nature and operational simplicity position it as a superior alternative to traditional radiosensitizers and thermotherapies.^17^ Additionally, LIUS has a greater tissue penetration depth than light‐based interventions.^18^ In recent years, significant progress has been made in understanding LIUS‐induced radiosensitization. However, a comprehensive understanding of the mechanisms driving radiosensitization through LIUS has not been well understood. To promote wider application of LIUS in RT, this review provided an overview of LIUS and its associated bioeffects in enhancing RT efficacy. Subsequently, potential mechanisms and application strategies for utilizing LIUS in radiosensitization were discussed.

## 2 MECHANISMS OF TUMOR RADIORESISTANCE

Tumor radioresistance remains a significant obstacle to successful treatment, mainly leading to local tumor remnants, recurrence, and metastasis, negatively impacting prognosis. Several foundational mechanisms contribute to tumor radioresistance, including DNA repair, signal transduction, hypoxia, and angiogenesis.^19^ Radiation causes a wide range of DNA damage, including double‐strand breaks (DSBs), single‐strand breaks (SSBs), base abnormalities, and protein cross‐linkages.^20^ The DNA repair system plays a crucial role in mending these damages, maintaining genomic stability and integrity. However, tumor cells can exploit their own repair genes and primarily undergo repair through homologous recombination or non‐homologous end joining (NHEJ) pathways.^21^

Two key signal transduction factors impacting radioresistance are NF‐kB and STAT3. NF‐kB plays a critical role in cell apoptosis. It remains inactive and can bind to the inhibitory protein IkB in typical circumstances. However, when cells are exposed to radiation, NF‐kB separates from IkB and moves into the nucleus, regulating the expression levels of various genes.^22^ NF‐kB regulates the expression levels of a wide array of genes involved in diverse biological processes, including inflammation, immunity, cell proliferation, and apoptosis. Some of the genes directly regulated by NF‐kB include those encoding pro‐inflammatory cytokines, such as tumor necrosis factor‐alpha (TNF‐α), interleukin‐1 (IL‐1), and interleukin‐6 (IL‐6), as well as chemokines, involving IL‐8. Additionally, NF‐kB controls the expression levels of anti‐apoptotic genes, such as Bcl‐2 and Bcl‐xL, promoting cell survival under stress conditions. Moreover, NF‐kB regulates the expression levels of genes involved in cell adhesion, angiogenesis, and tissue remodeling. The biological functions affected by NF‐kB activation are numerous and context‐dependent.^23^ In the context of radioresistance, NF‐kB activation can promote cell survival by upregulating anti‐apoptotic genes, thereby protecting cells from radiation‐induced cell death. Furthermore, NF‐kB‐mediated expression levels of pro‐inflammatory cytokines and chemokines can contribute to the recruitment of immune cells to the irradiated tissue, influencing the inflammatory response. In tumor cells, hyperactive NF‐kB impedes apoptosis, leading to heightened radioresistance.^24^ Conversely, STAT3, found to be overexpressed in a spectrum of tumor cells, is linked to tumorigenesis, progression, and malignancy, making it a promising target for tumor radiosensitization. STAT3 enhances aggressive tumor phenotypes through pathways, such as epithelial‐mesenchymal transition, radiation‐induced bystander effects, and alterations in the tumor microenvironment following RT. Moreover, STAT3 plays a role in DNA damage repair, further increasing tumor radioresistance.^25^

Tumor growth mainly leads to hypoxia due to insufficient oxygen reaching tumor cells through diffusion. Reactive oxygen species (ROS) generated during RT may cause tumor cell apoptosis. However, ROS effectiveness in causing oxidative stress is reduced in hypoxic environments, making tumor cells less susceptible to RT.^26^

RT also stimulates the expression level of vascular endothelial growth factor (VEGF), resulting in the enhanced angiogenesis and radioresistance. This process provides essential nutrients and oxygen required to support tumor growth, inadvertently undermining the effectiveness of RT.^27^ Angiogenesis inhibitors are deployed to disrupt angiogenesis, thereby cutting off the essential nutrients and oxygen supply to the tumor. This approach corrects abnormal angiogenesis by temporarily inhibiting the activities of proangiogenic factors. Using angiogenesis inhibitors can augment the responsiveness of tumor tissue to RT and elevate the death rate of tumor cells.^28^

## 3 MECHANISMS OF LIUS‐INDUCED RADIOSENSITIZATION

To induce radiosensitization, LIUS capitalizes on multiple mechanisms, such as inhibiting DNA repair, diminishing signal transduction, overcoming hypoxia, and obstructing angiogenesis. LIUS, as an emerging radiosensitization strategy, tackles tumor radioresistance by leveraging its bioeffects, usually comprising thermal, cavitation, and mechanical effects.

Thermal effects play a crucial role in the interaction between ultrasound beams and tissues.^29^ As the ultrasound beam passes through the target tissue, some of the ultrasonic energy is absorbed, and heat is generated.^30^ The amount of heat produced depends on various factors, including ultrasound parameters, tissue properties, and beam configuration.^31^

LIUS predominantly leverages cavitation effects due to its restricted thermal effects. To reduce the threshold needed to induce cavitation and strengthen the intensity of cavitation, ultrasound contrast agent microbubbles are often utilized. The formation and expansion of microbubbles filled with gas or liquid media leads to the occurrence of cavitation effects.^32^ Upon exposure to ultrasonic pressure, these microbubbles oscillate, causing vibration in the cell membrane. In the event of higher ultrasonic intensity, the microbubbles rupture, generating a powerful shockwave to harm the surrounding tissues.^33^ The effects of ultrasound cavitation are classified into two categories based on whether the microbubbles rupture. Both cavitation forms have significant physical, chemical, and biological impacts on tissues.^34^ In stable cavitation, microbubbles contract and expand repeatedly without rupturing, creating shear stresses between the microbubbles and cells that increase the permeability of cell membranes.^35^ Inertial cavitation occurs when microbubbles rupture under ultrasonic pressure, resulting in intense mechanical stresses, shock waves, acoustic streaming, free radicals, and high local temperatures. It causes irreparable damage to cells and tissue.^36^

**FIGURE 1:**
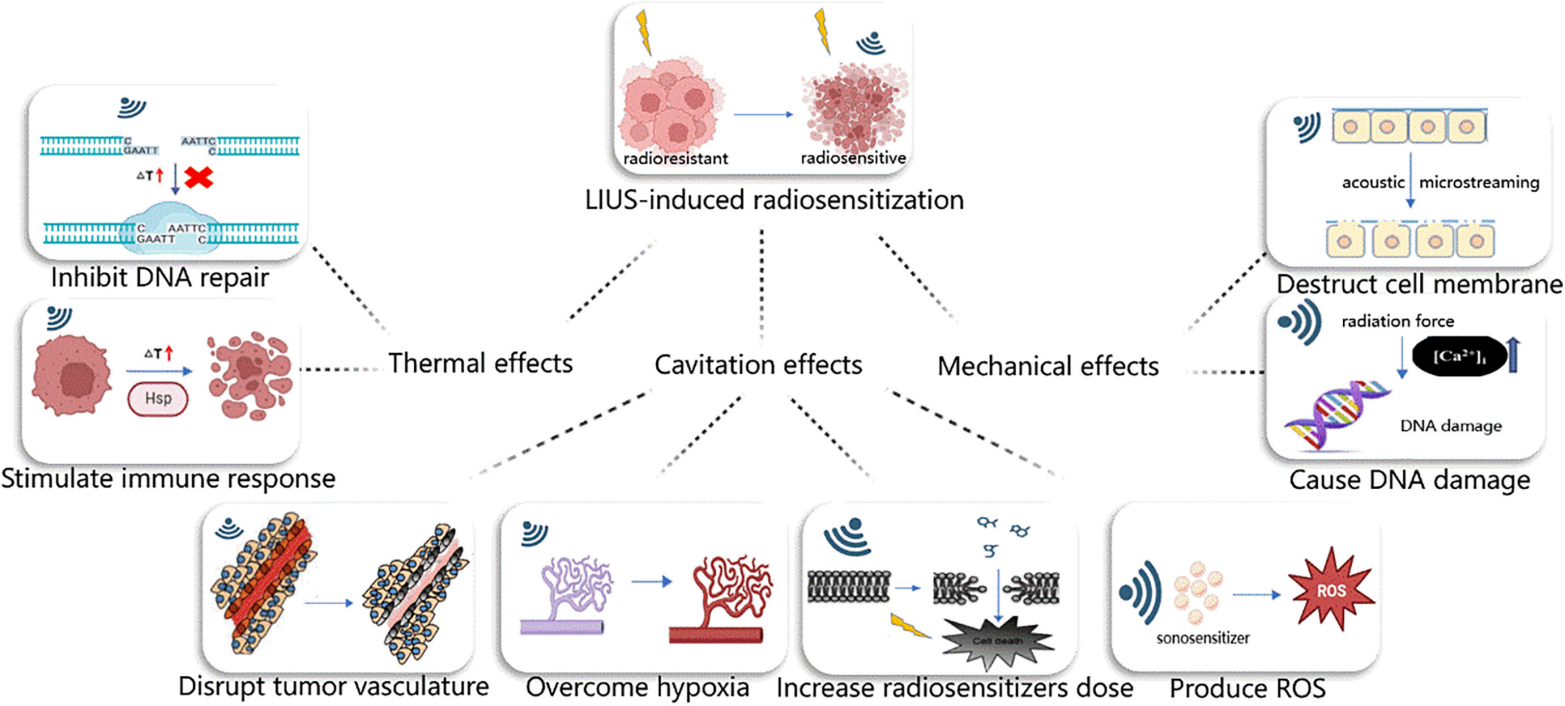
LIUS has bioeffects, including thermal, cavitation, and mechanical effects. Due to its bioeffects, LIUS induces tumor radiosensitization through various mechanisms.

In addition to the thermal and cavitation effects, the mechanical effects of LIUS are also crucial. Notably, the radiation force and acoustic microstreaming stand out as two primary mechanical effects.^37^ The ultrasonic beam exerts a radiation force, propelling objects in the path of wave propagation. This force aids in directing particles toward areas with the highest pressure amplitude.^29^ However, given its low‐intensity, the radiation force effects of LIUS are relatively limited.^38^ Acoustic microstreaming, on the other hand, is a consistent flow pattern initiated by the periodic radial oscillations of bubbles within a liquid medium. This intricate flow disrupts surrounding tissue structures, potentially leading to ruptures in cells or blood vessel walls.^39^ The mechanisms of LIUS‐induced radiosensitization through its bioeffects are summarized in Figure 1.

### 3.1 Promoting apoptosis of tumor cells

Apoptosis, an inherently programmed form of cell death, is a natural occurrence within living organisms. DNA damage repair significantly decreases radiation‐induced tumor cells apoptosis. Due to its thermal effect, LIUS causes local tissue heating. One potential side effect of using LIUS as a radiosensitizer is the risk of inducing tissue damage or injury due to the generation of heat. Although LIUS is typically used at low intensities to minimize thermal effects, there is still a possibility of local tissue heating, especially if higher intensities or prolonged exposure times are employed. This could lead to adverse effects, such as tissue necrosis, inflammation, or pain at the treatment site.^39^ In terms of treatment efficacy and safety compared to other radiosensitizers, such as cisplatin, LIUS possesses several potential advantages. LIUS can be targeted more precisely to the tumor site, allowing for localized sensitization of tumor cells to radiation while sparing surrounding healthy tissues. Additionally, LIUS can be applied non‐invasively and repeatedly without significant systemic toxicity, unlike some chemotherapeutic agents, such as cisplatin, which may cause systemic side effects, such as nephrotoxicity, neurotoxicity, or bone marrow suppression. However, the efficacy of LIUS as a radiosensitizer may vary depending on factors, involving the specific characteristics of the tumor, the ultrasound parameters used, and the timing of LIUS application relative to radiation therapy. Heat generated by LIUS inhibits homologous recombination, which is an essential process in repairing tumor DNA DSBs.^40^ While it is true that LIUS‐induced heat can inhibit homologous recombination, another DNA damage repair pathway that may be affected is NHEJ. NHEJ is a major pathway for repairing DNA DSBs and involves the direct ligation of broken DNA ends without the need for extensive homology. LIUS‐induced heat may disrupt the efficiency or fidelity of NHEJ by interfering with the proper alignment and joining of DNA ends, thus compromising the repair of DSBs.^41^ NHEJ involves a series of proteins, including Ku70/Ku80, DNA‐PKcs, XRCC4, and DNA ligase IV. These proteins act together to recognize and bind to DSBs, facilitate end processing, and mediate the ligation of broken DNA strands. Any disruption in the function of these proteins or the coordination of their activities could impair the NHEJ pathway. Therefore, LIUS is an effective way to inhibit the DNA damage repair of tumor cells and promote tumor cell death, which is of great significance to improve the RT's effectiveness.

Due to the transient cavitation induced by LIUS stimulation in cultured cells or solid tumors, the shock wave generated by the cavitation explosion activated sonosensitizers. The sonodynamic therapy (SDT) approach merges LIUS and sonosensitizers to promote the generation of ROS and trigger the subsequent molecular cascade reaction.^42^ SDT combines LIUS with sonosensitizers to induce transient cavitation, generating ROS in tumor cells. This ROS‐mediated cascade initiates oxidative stress, triggering a series of cellular signaling pathways, including MAPK, NF‐κB, and PI3K/Akt. The accumulation of ROS leads to lipid peroxidation, protein oxidation, and DNA damage, ultimately culminating in tumor cell apoptosis, autophagy, or necrosis. Moreover, SDT stimulates the immune system by releasing damage‐associated molecular patterns (DAMPs) and tumor‐associated antigens, promoting an antitumor immune response. Additionally, SDT exhibits antiangiogenic effects through the inhibition of angiogenesis‐related factors, such as VEGF. ROS produced by SDT eventually propel the apoptosis of cancer cells and advance the effectiveness of RT. In a study by Ma et al., a singlet oxygen fluorescence probe was used to detect ROS production in MCF‐7 breast cancer cells after SDT. A large amount of ROS production and the important role of ROS in inhibiting tumor growth has been found in their study.^43^ The mechanisms behind ROS production encompass sonoluminescence and pyrolysis.^44^ Sonoluminescence takes place when the inertial or stable cavitation of ultrasound generates light, which activates the sonosensitizers to create ROS. Pyrolysis involves inertial cavitation that raises the temperature and breaks down the sonosensitizer to produce free radicals. These free radicals then interact with other substrates to create ROS.^45^, ^46^

The mechanical effects of LIUS hold immense potential in oncological treatments and are increasingly recognized for promoting tumor cell apoptosis.^47^ Current research underscores that LIUS can bolster the efficacy of RT by triggering apoptosis in cancer cells through its mechanical effects.^48^ A study by Takashi Kondo and colleagues revealed that LIUS induces leukemic cell apoptosis, possibly by inflicting damage to the cell membranes via acoustic microstreaming.^49^ Shi et al. further explored the impact of LIUS on liver cancer cells, determining that LIUS can trigger apoptosis in hepatocellular carcinoma (SMMC‐7721 cell line) by regulating the Ca2+/mitochondrial pathway.^50^ Another noteworthy study by Matsuo et al. found that LIUS therapy considerably elevated the number of apoptotic cells in osteosarcoma LM8 cells. It has been demonstrated that LIUS curtails cell viability and impairs mitochondrial membrane potential, thereby triggering apoptosis.^48^

### 3.2 Stimulating immune response of tumor cells

There are immune mechanisms associated with the thermal effects of LIUS‐induced radiosensitization. One mechanism involves LIUS altering the fluidity of membrane lipids, resulting in a heat shock response without significant protein denaturation. This allows LIUS to initiate lipid remodeling and the expression of heat shock proteins (HSPs) while keeping cell membranes intact. LIUS irradiation leads to an increase in gene expression and cell surface localization of HSPs. When HSPs are stimulated in cell membranes, they become targets for natural killer cell‐mediated cytolysis, enhancing the radiosensitivity of tumor cells.^51^ Another potential mechanism involves the heat generated by LIUS inhibiting the activation of NF‐kB and STAT3, thereby reducing cellular radioresistance.^52^ It has been demonstrated that LIUS can block the phosphorylation of STAT3 induced by interleukin‐6, leading to the suppression of STAT3 expression.^53^ Additionally, LIUS treatment has been found to decrease the levels of proteins associated with the NF‐kB signaling pathway.^54^ It is important to note that LIUS has the expected radiosensitization effect with a low risk of burns due to the restricted thermal effects. This makes LIUS a promising approach for enhancing the effectiveness of RT while minimizing damage to normal tissues.

### 3.3 Overcoming hypoxia in tumor cells

Radiation exposure is harmful to tumor cells, while hypoxia provides them with a protective shield, inhibiting their death. Hypoxia frequently occurs in tumors due to inadequate blood supply. Consequently, an effective strategy to counteract hypoxia would significantly enhance the efficacy of RT.^55^ LIUS improves tumor tissue oxygenation by enhancing metabolism and blood perfusion through its thermal and cavitation effects.^40^ It has been demonstrated the promising potential of combining LIUS with microbubbles, which effectively enhances blood perfusion within solid tumors, thus overcoming tumor hypoxia. In rats with Walker‐256 tumors, UTMD demonstrated a remarkable improvement in tumor perfusion. Among potential explanations for this observed phenomenon is the cavitation effect of LIUS in conjunction with intravascular microbubbles. This might trigger inflammation that could rupture the microvessel wall and subsequently boost tumor blood flow. ELISAs performed on tumor tissue post‐UTMD treatment indicated the release of inflammatory vasodilator factors, such as eNOs, PGF2, and NO. These factors can temporarily augment blood flow and oxygen supply within the tumor, as evidenced further in a rabbit study where approximately 80% of the animal tumors manifested a substantial increase in blood perfusion after 10–20 min of UTMD treatment for VX2 tumors.^56^ Blood perfusion is significantly enhanced in poorly supplied tumors, and previously ischemic and nonperfused areas are reperfused.^57^ Recently, the mechanism by which UTMD enhances tumor perfusion has been studied by Zhang et al. in mice bearing MC38 colon cancers. The results demonstrated that UTMD resulted in tumor perfusion enhancement at MI = 0.3, and NO concentration increased at MI = 0.3/0.5 (*p* < 0.05).^58^

Oxygen microbubbles transport oxygen safely and efficiently in low‐oxygen environments. Upon ultrasound exposure, these microbubbles release additional oxygen, supplying oxygen to tumor cells dwelling in a low‐oxygen environment. Hypoxia inducible factor (HIF) governs the cellular response to hypoxia. Oxygenation increases and HIF expression decreases in the cytoplasm under the cavitation effect of LIUS upon oxygen microbubbles accomplish two objectives. First, it elevates oxygen levels within the tumor environment, and second, it curtails hypoxic signals.^59^

### 3.4 Inducing sonoporation in tumor vascular endothelial cells

There are numerous mechanisms through which the cavitation effects of LIUS sensitize tumor cells to radiation. For instance, sonoporation creates tiny pores in cell membranes that induce apoptosis of tumor vascular endothelial cells or accelerate the transport of radiosensitizers.^11^ Tumor vascular endothelial cells are the initial target of microbubbles driven by ultrasound.^60^ LIUS combined with intravascular microbubbles results in cavitation effects that trigger ceramide synthesis in tumor vascular endothelial cells.^61^ While ceramide synthesis cannot directly overcome radioresistance in tumor cells, it harms tumor vascular endothelial cells through ceramide‐dependent mechanisms triggered by the acid sphingomyelinase (ASMase) signal.^62^ When UTMD is combined with RT, a synergistic effect occurs, leading to tumor vascular endothelial cell death and vascular collapse.^63^ According to a recent study, fibrosarcoma mice with UTMD+RT treatments increased the signal for ceramide‐induced tumor cell death. Furthermore, tumors in ASMase−/−mice exhibited radioresistance to UTMD+RT treatments. It confirmed the activation of ASMase during UTMD‐enhanced vascular disruption in RT.^64^ Another study conducted on prostate cancer PC3 cells revealed that suppression of UGT8 leads to a surge in ceramide levels and a decline in the vascular index. Downregulated UGT8 had a higher degree of harm inflicted on the tumor cells compared to the control cells. Conversely, tumor cells with upregulated UGT8 exhibited a lower mortality rate than control cells. These findings suggest that the accumulation of ceramide, owing to activation of ASMase and downregulation of UGT8, triggers amplified cell death signals and enhances the efficacy of RT.^65^

By utilizing LIUS combined with microbubbles, the ensuing cavitation effects can generate shock waves with the capability of penetrating cell membranes. This occurrence, termed sonoporation, imparts temporary and reversible damage to cells or tissues.^66^ Additionally, the cavitation effects lead to the production of free radicals. These radicals can oxidize the phospholipid bilayer of the cell membrane, thus amplifying its permeability.^67^ In a study conducted by Mei et al., it was revealed that the introduction of microbubbles into normal rat blood circulation and subsequent irradiation of the femoral artery with LIUS (1.0 MHz, 0.22 MPa) led to the flow of calcium ions and release of dextran into the extracellular space, thereby confirming the creation of micropores in the vascular endothelial cell membrane.^68^ Furthermore, when the permeability of the “blood‐tumor barrier” is temporarily heightened, the delivery of drugs to tumor tissue can be markedly optimized. The sonoporation mechanism induced by LIUS can disrupt the integrity of the “blood‐tumor barrier.”^69^ In a study by Chen et al., UTMD was employed on the hippocampus region of mouse brains. Results indicated that the blood–brain barrier in the irradiated zone was uniformly broken at a sound pressure of 0.45 MPa.^70^ Such effects pave the way for more effective delivery of radiosensitizers, increasing the dosage that reaches tumor cells.

## 4 APPLICATION STRATEGIES OF LIUS IN RADIOSENSITIZATION

Recent progress in ultrasound technology has presented exciting opportunities for leveraging LIUS bioeffects to induce radiosensitization. A variety of application strategies of LIUS in radiosensitization have been explored, such as LIUS alone, ultrasound‐targeted microbubble destruction (UTMD), ultrasound‐mediated radiosensitizers delivery and SDT. LIUS alone predominantly utilizes the thermal and mechanical effects of LIUS, while other strategies concentrate on cavitation and mechanical effects. Table 1 offers an overview of the experimental studies conducted on LIUS‐induced radiosensitization utilizing different strategies.

**TABLE 1:** Research progress of LIUS‐induced tumor radiosensitization.

Strategies	Tumor cells	Parameters	Results	Potential principles	Reference
LIUS alone	Human myelomonocytic leukemia cells (U937) and human acute lymphoblastic leukemia cells (Molt‐4)	0.3 W/cm^2^ 1.0 MHz	LIUS enhanced the anticancer effects of radiation	LIUS‐induced reversible membrane damage or ionic transport across the membrane	[71]
	B16‐F1 melanoma cells (mouse)	3 W/cm^2^ 1.0 MHz	LIUS enhanced RT‐induced delay in tumor growth	LIUS‐induced lipid remodeling and heat shock protein stress	[72]
	TPSA23 prostate tumor cells (mouse)	3 W/cm^2^ 1.0 MHz	LIUS+RT achieved a more complete response of tumors than RT alone	LIUS reduced STAT3‐mediated cellular radiation resistance	[53]
UTMD	Human umbilical vein endothelial cells (HUVEC), acute myeloid leukemia cells (AML), murine fibrosarcoma cells (KHT‐C), prostate cancer cells (PC3), breast cancer cells (MDA‐MB‐231) and astrocytes cells	570 kPa 0.5 MHz	RT‐induced cell death was enhanced by exposing cells to UTMD	UTMD caused sufficient ceramide production to cause cell death	[73]
	Human esophageal carcinoma cells (KYSE‐510) and HUVECs	570 kPa 1.0 MHz	UTMD promoted RT‐induced apoptosis in esophageal carcinoma cells	UTMD inhibited angiogenesis and reduced esophageal carcinoma cell proliferation	[74]
	Prostate cancer cells (PC3)	240 kPa 0.5 MHz	UTMD enhanced the efficacy of RT	UTMD downregulated UGT8 that leads to ceramide accumulation and initiated an apoptotic signal	[65]
	Human prostate cancer cells (mouse)	570 kPa 0.5 MHz	UTMD enhanced the RT effects	UTMD caused ceramide accumulation and was involved in responses reduced vessel density	[75]
	Human bladder cancer HT‐1376 cells (mouse)	0.8 MI 570 kPa 0.5 MHz	UTMD enhanced radiation effects in tumor, and led to enhanced tumor cell death	UTMD caused vascular endothelial cell disruptions and direct tumor cell kill	[76]
	Human breast cancer MDA‐MB‐231 cells (mouse)	0.8 MI 570 kPa 0.3 MHz	UTMD enhanced the effects of radiation	UTMD disrupted tumor vasculature and reduced vessel density	[77]
	Human breast cancer MDA‐MB‐231 cells (mouse)	570 kPa 0.5 MHz	UTMD sensitized tumors to RT	UTMD caused localized vascular effects and reduced vessel density	[78]
	Human hepatocellular carcinoma cells (rat)	2.5 MPa 4.2 MHZ	UTMD sensitized tumor tissue to RT and improved outcomes	UTMD induced vascular endothelial cell apoptosis	[79]
	Fibrosarcoma MCA/129 cells (mouse)	0.8 MI 500 kPa 0.5 MHz	UTMD enhanced RT response in tumor	UTMD activated ASMase‐ceramide pathway in vascular targeting	[80]
	Human glioblastoma cells (U87MG) (mouse)	0.3 W/cm^2^ 1 MHz	UTMD enhanced RT destructive effect of glioblastoma	UTMD caused blood vessel disruption and tumor tissue impairment	[81]
	Human prostate cancer cells (mouse)	0.8 MI 0.5 MHz	UTMD+XRT significantly decreased sizes of tumors	UTMD created a vascular response and disrupted tumor vasculature	[82]
	Human prostate cancer cells (rabbit)	565 kPa 0.5 MHz	UTMD+RT showed a superior antitumor effect than RT alone	UTMD caused tumor vascular disruption and reduced vessel density	[83]
	Breast cancer tumors cells (mouse)	1.39 MI 2.5 MPa 4.2 MHz	UTMD overcame radioresistance in hypoxic tumor volumes	Oxygen microbubbles increased the oxygenation level	[84]

	Head and neck squamous cell carcinoma (HNSCC) cells (mouse)	1.4 MI 4 MHz	UTMD improved radiosensitivity and animal survival in an HNSCC model	Oxygen microbubbles improved tumor energetics and oxygenation prior	[85]
	Human breast cancer MDA‐MB‐231 cells (mouse)	2 W/cm^2^ 1 MHz	UTMD enhanced radiosensitivity in MDA‐MB‐231 tumor‐bearing mice	UTMD gave rise to oxygen release from Oxygen microbubbles targeted tumor	[86]
	4 T1 breast cancer cells (mouse)	2 W/cm^2^ 1 MHz	UTMD can significantly enhance the effects of RT	Oxygen microbubbles delivered oxygen to tumor tissue	[87]
Hepatocellular carcinoma (HCC) cells (patient)	1.13 MI 1.5 MHz	UTMD improved hepatocellular carcinoma TARE treatment response	UTMD localized insult to the vascular endothelial cells	[88]
SDT	HeLa cells	1or1.5 W/cm^2^ 1or2 MHz	SDT improved the radiosensitization effect of LIUS to 9.93 times	SDT made tumor lesions absorb more ultrasonic beams and produce more ROS	[89]
	Breast cancer cells (mouse)	0.1or2W/cm^2^ 1or2 MHz	SDT enhanced the RT effectiveness	SDT produced a large number of ROS	[90]
	HeLa cells	0.4 W/cm^2^ 1 MHz	LIUS and radioenhancer exhibit a synergistic effect in TSER	LIUS targeted tumor cells to stimulate sonosentisizers producing ROS	[91]
	GBM cells	0.4 W/cm^2^ 1 MHz	LIUS and radioenhancer exhibit a synergistic effect in TSER	LIUS targeted tumor cells to stimulate sonosentisizers producing ROS	[92]

### 4.1 LIUS alone

High‐intensity ultrasound (HIUS) can significantly enhance the effectiveness of RT by sensitizing cancer cells to radiation through thermal effects. In a clinical study conducted by Wu et al., it has been found that the combination of HIUS and RT resulted in higher overall survival rates in prostate cancer patients than RT alone.^93^ However, HIUS may cause damage to normal tissues due to rapid heating. LIUS has a smaller thermal effect and can transmit sound energy to tumor tissue without causing harm to normal tissue.^94^

LIUS offers advantages in terms of safety, precision, controllability, and strong penetrating force. It has been observed that LIUS decreases tumor radioresistance by utilizing its thermal and mechanical effects.^72^ LIUS amplifies radiation‐induced tumor cell death under noninertial cavitational conditions. The radiosensitization capacity of LIUS has been confirmed in various cell and animal experiments. For example, when human myelomonocytic leukemia cell lines (U937) and human acute lymphoblastic leukemia cell lines (Molt‐4) were exposed to LIUS shortly after radiation, it was found that LIUS has the potential to enhance the anticancer properties of radiation.^71^ A study conducted on mice with B16 melanoma, it also shown a promising result. The mice were divided into three groups: LIUS, RT, and LIUS+RT. Tumor volume was measured regularly, and it has been demonstrated that the tumors in the LIUS group continued to grow, while the tumors in the RT and LIUS+RT groups exhibited significant growth delays within 3 weeks of treatment.^72^ Additionally, mice treated with RT alone showed tumor regrowth, whereas the LIUS+RT group displayed limited tumor growth and even complete tumor regression. It has been found that LIUS can effectively control the growth of established melanomas by increasing sensitivity to radiation.^51^

Although there are no related clinical trials, the safety and controllability of LIUS‐induced radiosensitization make it a significant area for clinical research and application. It is crucial to optimize parameters, such as ultrasound frequency, intensity, and irradiation time. The thermal effects of LIUS are closely related to these parameters and result in normal tissue damage if not carefully controlled. Therefore, finding the best acoustic parameters is essential to achieve optimal radiosensitization effects on tumors.

### 4.2 Ultrasound‐targeted intravascular microbubbles destruction

Microbubbles are tiny bubbles filled with gas that are less than 10 μm in diameter. They are commonly used as contrast agents in ultrasound imaging.^95^ The use of microbubbles has expanded the capabilities of ultrasound imaging to include molecular imaging and targeted therapies. Compared to LIUS alone, UTMD possesses numerous advantages, such as increased target specificity and efficiency.^96^ It has been demonstrated that the efficacy of LIUS‐targeted intravascular microbubbles destruction in antiangiogenesis. The combination of UTMD and RT was effective across various cell lines, such as HUVECs, AML cells, KHT‐C cells, PC3 cells, MDA‐MB‐231 cells, and KYSE510 cells. These findings indicate a collaborative effort between UTMD and RT to inhibit angiogenesis by decreasing the survival rates of endothelial cells.^73^, ^74^ In animal models of breast, colon, and liver cancers, UTMD has been found to contribute significantly to tumor vascular destruction.^97^, ^98^, ^99^

Gregory Czarnota and his colleagues conducted a series of studies to investigate the potential vascular effects induced by UTMD and whether they could lead to radiosensitization. The researchers conducted their experiments using mice that had PC3 prostate cancer xenografts. They administered microbubbles intravenously to the mice and used LIUS to stimulate the microbubbles, which caused disruptions in the endothelial cells within the tumor vessels. The study involved three groups of mice that were used for noninvasive studies of acute effects, longitudinal effects, and blood flow, in addition to a control group. The researchers designed nine different experimental conditions consisting of combinations of three factors: the presence or absence of microbubbles, low or high concentration of microbubbles activated by ultrasound, and a one‐time radiation dose of 0, 2, or 8 Gy. This allowed them to evaluate the effects of varying radiation doses and microbubble concentrations on vascular closure and cell death. It has been demonstrated that combining UTMD with radiation led to a significant increase in vascular closure and cell death within the tumor. A synergistic effect between radiation and UTMD has been observed, indicating that the combination of the two treatments had a greater impact compared to using each treatment alone. Specifically, the combination of 2 Gy radiation and a high microbubble concentration resulted in 44.13% cell death, while the combination of 8 Gy radiation and a high microbubble concentration resulted in 70.8% cell death.^75^ It provided a previously undescribed report of UTMD being used to enhance radiation effects. In addition to the PC3 prostate cancer xenograft model, similar findings were also observed in mouse and rat models of bladder cancer, breast cancer, hepatocellular carcinoma, fibrosarcoma, and glioblastoma. There is no doubt that UTMD‐induced vascular closure and tumor cell death have potential applications in enhancing the RT's effectiveness.^76^, ^77^, ^78^, ^79^, ^80^, ^81^

**FIGURE 2:**
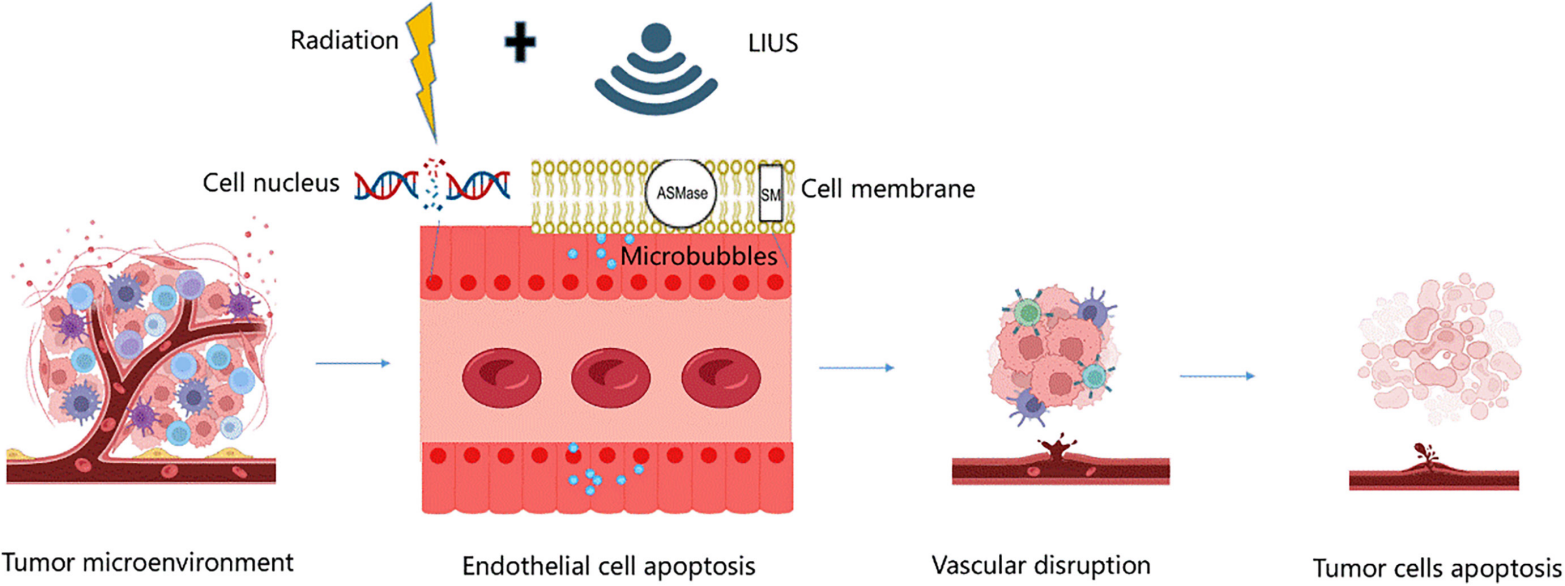
UTMD destroys tumor vascular endothelial cells to induce radiosensitization. UTMD causes ceramide‐dependent mechanisms induced by acid sphingomyelinase (ASMase) signaling in endothelial cell membranes. Radiation causes DNA damage in the endothelial cell nucleus. UTMD results in more cell death signals and greater enhancement of radiation effects.

Researchers have further conducted investigations into the effects of UTMD‐induced radiosensitization on larger tumor models in rabbits with prostate tumor xenografts. The results proved that that combining UTMD and RT resulted in enhanced radiological effects and improved antitumor efficacy when compared to using each therapy alone.^83^ In a recent study, the combination of UTMD and fractionated low‐dose RT was further explored. In this study, mice were divided into separate groups for fractionated RT alone and fractionated RT combined with UTMD. Over the course of a 4‐week treatment period, the combination group exhibited a significant decrease in tumor size and reduced blood flow within the tumor. In contrast, the group receiving fractionated RT alone experienced an increase in tumor size.^82^ LIUS activates a ceramide‐dependent mechanism via the acid sphingomyelinase (ASMase) signal through cavitation has been indicated in animal studies. This mechanism leads to the destruction of tumor vascular endothelial cells and subsequently induces radiosensitization^80^ (Figure 2).

While preclinical studies have provided substantial evidence of the potential therapeutic benefits of combining UTMD with RT, clinical trials are essential to demonstrate its feasibility in human patients. A clinical study conducted by Gummadi et al. utilized UTMD in conjunction with transarterial radioembolization (TARE) for primary hepatocellular carcinoma (HCC). The study involved 28 HCC patients and revealed that the effectiveness of UTMD combined with TARE was significantly higher than that of TARE alone.^88^ This demonstrates great promise in the safety of adding UTMD to existing radiation protocols.^100^ Both basic and clinical studies have shown significant radiosensitization effects and improved survival rates and quality of life in experimental animals or patients.^83^ The cavitation effect of LIUS not only damages the integrity of tumor blood vessels but also significantly reduces the adverse effects of radiation, making RT more effective and safer.^81^ This approach holds the potential for enhancing the therapeutic outcomes of cancer treatment. However, further research needs to explain the mechanisms involved and to validate its efficacy in clinical settings.

### 4.3 Ultrasound‐mediated targeted radiosensitizers delivery

Ultrasound‐mediated targeted drug delivery is an emerging method for noninvasive and targeted enhancement of drug uptake. Its cavitation effects make it a candidate to trigger radiosensitizers delivery.^101^ Ultrasound‐mediated radiosensitizers delivery involved either coadministration of microbubbles with radiosensitizers or conjugating radiosensitizers onto microbubbles followed by exposure to ultrasound. One clinical method that has been proven effective is the use of chemotherapeutic agents as radiosensitizers. Paclitaxel and gemcitabine are two such agents that have demonstrated radiosensitizing activity.^102^ Although agents can enhance tumor radiosensitivity, their side effects can limit patient eligibility and treatment efficacy, ultrasound‐mediated targeted radiosensitizers delivery reduces the normal‐tissue toxicity of agents.^103^ In one study, a 1 MHz single‐element ultrasound transducer was used to investigate the effectiveness of this approach in delivering nab‐paclitaxel to a pancreatic tumor model. It has been demonstrated that using microbubble‐assisted ultrasound in combination with nab‐paclitaxel increased drug delivery and improved therapeutic effectiveness over nab‐paclitaxel treatment alone in both in vivo and in vitro experiments.^104^ In another study, LIUS at 3 MHz and 0.26 MPa was administered to stimulated microbubbles before, during, or after gemcitabine infusion. It has been found that LIUS‐stimulated microbubbles increased the gemcitabine concentration in rabbit VX2 tumors, with the highest drug concentration observed in the group that received LIUS‐stimulated microbubbles treatment immediately after gemcitabine infusion. This group had a drug concentration in tumors that was 2.83 times higher than that of the control group.^105^

Logan et al. developed a method to create microbubbles loaded with the chemotherapy drugs gemcitabine and paclitaxel, helping to enhance RT's effectiveness.^106^ Meanwhile, Bhardwaj et al. designed a special nano‐system that helps deliver curcumin, a plant compound, along with paclitaxel, to treat locally advanced triple‐negative breast cancer (TNBC). They found that focusing ultrasound on the tumor helps these nanoparticles enter the tumor faster, safely, and more efficiently than traditional paclitaxel treatment. Moreover, including curcumin with paclitaxel in these nanoparticle delivery systems improves RT's effectiveness. These findings highlight the potential for using curcumin, paclitaxel, radiation, and ultrasound in combination to treat various types of tumors that respond to radiation.^107^ As research continues in this area, it is hoped that more will be discovered about the potential of LIUS targeted therapy in enhancing radiosensitizer concentration for various chemotherapy drugs and tumors.

Tumor hypoxia can result in tumor radioresistance. Oxygen has been of great interest as a radiosensitizer. Oxygen microbubbles are used to reverse oxygen depletion and improve radiosensitization, oxygen delivery induced by ultrasound irradiation relieves hypoxia instantly. In a rat fibrosarcoma model study by Paul et al., oxygen microbubbles improved RT effects were demonstrated for the first time.^108^ It has been demonstrated that oxygen microbubbles significantly increase the oxygenation level of breast cancer in mouse models, leading to a delay in tumor growth when combined with RT.^109^, ^110^, ^111^ The filling gas of the microbubbles is modified by the addition of sulfur hexafluoride to oxygen such that the obtained oxygen/SF6 microbubbles (OS MBs) achieve a much longer half‐life (>3×) than that of oxygen microbubbles. The OS MBs are tested in nasopharyngeal carcinoma (CNE2) tumor‐bearing mice and oxygen delivery by the OS MBs induced by ultrasound irradiation has notable effects in reversing hypoxia and radiosensitization.^84^ In a mouse head and neck squamous cell carcinoma tumor model, a novel ultrasound‐sensitive microbubble loaded with oxygen and a pharmacological inhibitor of tumor mitochondrial respiration (lonidamine (LND)) was used to successfully sensitize tumors to radiation.^85^ Ultrasound‐sensitive microbubbles loaded with oxygen and LND provided prolonged oxygenation relative to oxygenated microbubbles alone, as well as provided an ability to locally deliver LND, making them more appropriate for clinical translation.^86^ While these studies were conducted in immunocompromised mice, future work will focus on larger immune‐normal animals to evaluate the clinical relevance of the platform. The hope is to extend this radiosensitization strategy to other solid tumors.

### 4.4 Sonodynamic therapy

**FIGURE 3:**
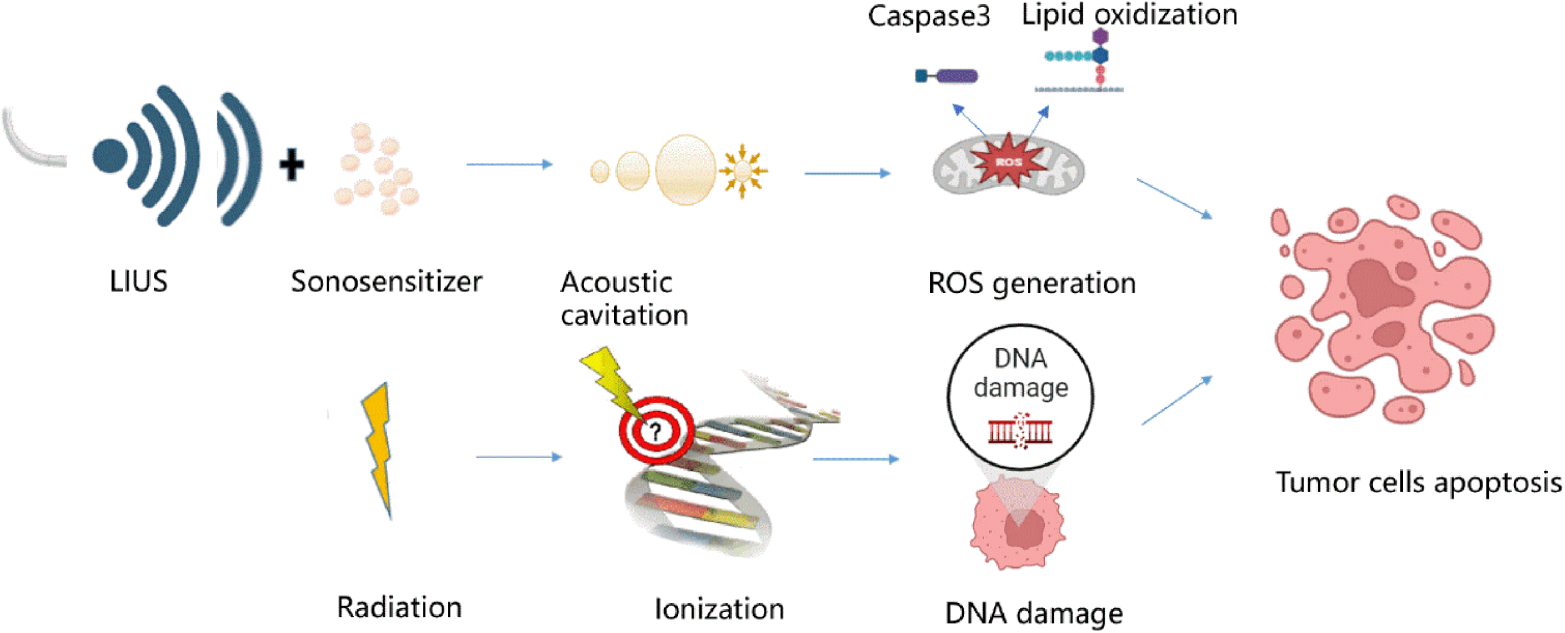
Complementarity of SDT and RT. In the presence of a sonosensitizer, LIUS causes cavitation, leading to ROS generation. ROS promotes the expression of caspase‐3 through mitochondrial destruction and lipid oxidization in cell membranes with resultant cell death by apoptosis and necrosis. RT only causes DNA double‐strand breaks in the cell nucleus, so SDT is a powerful supplement to RT.

SDT is a novel tumor treatment method with low invasiveness and high precision.^87^ While it originated from photodynamic therapy (PDT), the basic principle of SDT differs slightly from that of PDT. In the past, the RT‐PDT combination has been appealing because RT and PDT cause cancer cell death through different mechanisms. RT induces cell apoptosis through DNA damage in the cell nucleus, while PDT usually results in traditional photosensitizers accumulating in cell membranes and mitochondria, thus inducing cell apoptosis in these sites.^112^ RT‐induced gene mutation often prevents tumor cells from initiating apoptosis in response to irreparable DNA damage, leading to radioresistance,^113^ so PDT is a powerful supplement to RT.^114^ The results of multiple clinical studies supported that the PDT‐RT combination is more beneficial than RT in lymphoma, lung cancer, and esophageal cancer.^115^, ^116^, ^117^ Despite the benefits of PDT, it is plagued by two disadvantages: weak tissue penetration and skin phototoxicity of photosensitizer absorption.^118^ Fortunately, SDT avoids these two PDT problems. Compared to PDT, SDT provides numerous opportunities and benefits, including deeper tissue penetration, higher precision, fewer side effects, and better patient compliance.^119^ SDT can also promote lipid oxidization in cell membranes and the expression of caspase‐3 through mitochondrial destruction and eventually promote tumor cell apoptosis (Figure 3). There is no doubt that SDT, as a new radiosensitization strategy, is better than PDT.

Exploring the effects of different doses of ionizing radiation (IR) on tumor sensitivity is crucial for understanding the underlying mechanisms and optimizing treatment strategies. Various mechanisms contribute to tumor sensitization at different radiation doses, including DNA damage, cell cycle arrest, apoptosis, and modulation of the tumor microenvironment. At lower doses, IR may induce sublethal DNA damage, leading to cell cycle arrest or repair mechanisms that promote cell survival. However, this can also sensitize tumors to subsequent radiation fractions by impairing DNA repair pathways, increasing genomic instability, and enhancing the efficacy of subsequent radiation doses.^120^ At higher doses, IR can directly induce lethal DNA damage, leading to cell death via apoptosis or mitotic catastrophe. Additionally, higher doses of radiation may trigger complex signaling cascades in the tumor microenvironment, leading to inflammation, vascular damage, and changes in tumor oxygenation that further enhance tumor radiosensitivity.^121^

SDT‐induced radiosensitization was first demonstrated in an in vitro experiment. Gold nanoparticles, as sonosensitizers, are deposited in tumor tissues through enhanced permeability and retention so that tumor tissues absorb more ultrasonic beams and produce more ROS. To investigate the sonodynamic‐radiosensitivity effect of gold nanoparticles, cervical cancer HeLa cells were treated differently by RT, LIUS, and RT+LIUS irradiation and then combined with different concentrations of gold nanoparticles with RT+LIUS. The effects of different treatments on HeLa cell survival rates were evaluated, and the results showed that LIUS can enhance RT effectiveness and that SDT with the application of LIUS and gold nanoparticles improved the radiosensitization effect of LIUS to 9.93 times.^89^ However, the further potential clinical translation of gold nanoparticles in x‐ray and US theranostics might be limited by their low biodegradation rate and potential toxicity. Ye et al. further carried out an animal experiment and designed a two‐step strategy based on PP18‐Pt NPs. The first step is to relieve tumor hypoxia by LIUS. In the second phase, PP18‐Pt NPs synthesized from polyethylene glycol (PEG), the radiosensitizer platinum, and the sonosensitizer PEG‐Purpurin 18 were injected into mice inoculated with 4T1 breast cancer cells. The results showed that the SDT‐RT combination produced more ROS than RT alone to enhance the intensity of oxidative stress injury and inhibit the growth of 4 T1 breast cancer cells.^90^ These studies indicated that SDT produces a large number of ROS to induce radiosensitization, and its effects are better than those of LIUS alone.

A sonosensitizer chlorin e6 (Ce6) was used by Chen et al., which could be activated by ultrasound and specifically delivered to mitochondria of tumor cells. A folic acid (FA)‐conjugated carboxymethyl lauryl chitosan (CLC)/superparamagnetic iron oxide (SPIO) micelle was developed to precisely deliver hydrophobic Ce6 to HeLa cells. This was followed by LIUS after which only the Ce6‐internalizing HELA cells received the LIUS‐induced Ce6 sensitization that might show lower radioresistance. Then, low‐dose radiation, that was not harmful to normal cells, was employed to exert highly tumor cell‐specific toxicity. This newly developed tumor cell‐targeted RT was named TSER, which has rarely been reported and merits systematic investigation in their research. Subsequently, it has been demonstrated that the Ce6‐receiving HELA cells would be significantly killed after the synergistic effect from LIUS and low‐dose radiation.^91^ Building on this foundation, Chiang et al. facilitate GoldenDisk (a radioenhancer) in TSER. By combining GoldenDisk with LIUS and a sonosensitizer 5‐aminolevulinic acid, GBM cells were sensitized to RT leaving healthy tissues in the vicinity unaffected. When GBM cells underwent TSER treatment, a significant reduction in cell viability was observed.^92^ Such a combinational therapeutic paradigm has suggested novel insights into the application of sonosensitizer‐based synergistic cancer sonoradiotherapy, which provides a more feasible, readily available cancer therapeutic option.

It has been demonstrated the potential for SDT‐induced radiosensitization through the generation of ROS in previous research involving in vitro cultured cells and small animal models. While there has been significant progress in SDT‐induced radiosensitization, it is still in the early stages of development, and further studies are necessary before clinical application. First, a deeper understanding of the SDT‐induced radiosensitization mechanisms is crucial. This understanding will lay the foundation for the development of more effective combination therapies. Second, rigorous evaluation of the biosafety of sonosensitizers is needed through multiple studies before considering their clinical application.^45^

## 5 CONCLUSION AND PROSPECTS

LIUS is a widely utilized imaging technique renowned for its safety and convenience in diagnosing diseases. Notably, LIUS employs cost‐effective equipment to foster tumor radiosensitization, curtail radiation exposure, and elevate the quality of life for patients. This efficacy is attributed to LIUS's ability to utilize thermal, cavitation, and mechanical effects to overcome tumor cell resistance to RT. Multiple experimental analyses have underscored the effectiveness of LIUS in inducing tumor radiosensitization using diverse strategies, including LIUS alone, ultrasound‐targeted intravascular microbubble destruction, ultrasound‐mediated targeted radiosensitizers delivery, and sonodynamic therapy.

As a relatively new method of radiosensitization, there are still some unknowns about the underlying mechanisms of LIUS that require further investigation. It is also essential to customize treatment parameters for different tumor types and locations. While initial studies have shown promising results, conducting more comprehensive clinical trials is crucial to confirm its safety and effectiveness in real‐world situations. These ongoing clinical studies are essential in advancing the application of LIUS as a groundbreaking approach for improving human health.

## AUTHOR CONTRIBUTIONS

**Haonan Xu:** Data curation (lead); formal analysis (lead); methodology (lead); writing – original draft (lead). **Zichao Liu:** Data curation (lead); methodology (lead); writing – original draft (lead). **Meng Du:** Funding acquisition (lead); project administration (lead); supervision (lead); writing – review and editing (lead). **Zhiyi Chen:** Conceptualization (lead); funding acquisition (lead); project administration (lead); resources (lead); supervision (lead); writing – review and editing (equal).

## FUNDING INFORMATION

This study was supported by the National Natural Science Foundation of China (Grant No. 82272028, 81971621, and 82102087), the Key R&D Program of Hunan Province (Grant No. 2021SK2035), the Natural Science Foundation of Hunan Province (Grant No. 2022JJ30039 and 2022JJ40392), and the Project of Science and Technology Innovation of Hunan Province (Grant No. 2021SK51807).

## CONFLICT OF INTEREST STATEMENT

The authors declare that there is no conflict of interest.

## Data Availability

Data sharing is not applicable to this article as no new data were created or analyzed in this manuscript.
